# Strengthening microbial genomics capacity in Africa for epidemic preparedness: key lessons from the AFROSCREEN programme

**DOI:** 10.7189/jogh.16.03012

**Published:** 2026-04-30

**Authors:** AFROSCREEN consortium, Salissou Abdou Ahmed Mohamed, Steve Ahuka-Mundeke, Prince Akil, Yoan Alaho, Mathias Altmann, Raphael Amani, Marie Amougou Atsama, Adrienne Amuri Aziza, Soa Fy Andriamandimby, Reine Salomé Anguinze Yegbia, Eloïc Lénox Atindegla, Ahidjo Ayouba, Alexandre Azar, Aliou Barry, Dieynaba Barry, Naima Barry, Alix Blévin, Isabelle Bonal, Tara Brosschot, Valérie Caro, Margaux Chavardes, Bakary Cisse, Fatoumata Cisse, Adjo Seyram Comlan, Cathy Sandra Goimelle Coti-Reckoundji, Gilles Cottrell, Caroline Coulon, Anoumou Claver Dagnra, Muriel De Souza, Eric Delaporte, Koussay Dellagi, Lamine Dia, Ndongo Dia, Moussa Moise Diagne, Fatoumata Diallo, Haby Diallo, Mamadou Korka Diallo, Maryam Diarra, Fatoumata Diawara, Fatoumata Dicko, Idrissa Dieng, Seynabou Mbaye Ba Souna Diop, Delia Doreen Djuicy, Eric D'Ortenzio, Natasha Dubois Cauwelaert, Ginette Elvine Edoul Mbesse, Marion Fanjat, Ella Cyrielle Farra née Gonofio, Ousmane Faye, Nicolas Fernandez-Nuñez, Nadine Fievet, Sandra Miriella Charlène Garba-Ouangole, Thibaut Armel Chérif Gnimadi, Célestin Godwe, Solène Grayo, Emilande Guichet, Sylvie Guillemaut, Ibrehima Guindo, Juliette Hardy, Alice Henry-Tessier, Magali Herrant, Parfait Houngbegnon, Roughyatou Ka, Adèle Kacou-N'Douba, Kadio Jean-Jacques Olivier Kadio, Hervé Kadjo, Aliou Kamissoko, Mouhamed Kané, Dramane Kania, Ouattara Yakoura Karidja, Alpha Kabinet Keita, Eddy Kinganda Lusamaki, Stéphane Kouadio Koffi, Kobina Amandze Adams Kofi, Giscard Francis Komoyo, Drissa Kone, Yekayo Bénédicte Kone-Dakouri, Abla Ahouefa Esther Konou, Yao Rodion Konu, Charles Kouanfack, Vincent Lacoste, Adamou Lagare, Magali Lago, Ramatoulaye Lazoumar, Yann Le Pennec, Cheikh Loucoubar, Estelle Madaha, Martin Maidadi-Foudi, Aminata Maiga, Christian Malaka, Santou Mamadou, Alexandre Manirakiza, Achille Massougbodji, Placide Mbala-Kingebeni, Maimouna Mbanne, Aminata Mbaye, Geraldine Meyer, Sarah Michel-Anfray, Serge Freddy Moukaha-Doukanda, Clara Müller, Joyce Mwongeli Ngoi, Mouchedou Abdoukarim Naba, Ousseynou Ndiaye, Christelle Nikiema, Richard Njouom, Odilon Paterne Nouatin, Justus Nsio, Dieudonnée Ouedraogo, Armelle Pasquet, Martine Peeters, Julien Poublan, Nicole Prada, Claudio Raharinandrasana, Vololoniaina Raharinosy, Christian Ranaivoson, Rindra Randremanana, Tsiry Hasina Randriambolamanantsoa, Rila Ratovoson, Norosoa Razanajatovo, Vincent Richard, Pierre Roques, Céline Rouger, Mounerou Salou, Safietou Sankhe, Rabiatou Sanogo, Yacouba Sawadogo, Maud Seguy, Islamiath Setondji Kissira, Emeline Simon, Rachida Tahar, Aristophane Tanon, Jules Brice Tchatchueng Mbouga, Mathurin Tejiokem, Mame Salane Thiam, Isaac Tiembre, Bachirou Tinto, Noël Tordo, Abdoulaye Toure, Coumba Toure Kane, Isidore Traore, Noumou Yacouba Keita, Anges Yadouleton, Brice Yambyo, Abo Yao, Guillaume Zamina, Adama Zida, Arsene Zongo

## Abstract

The AFROSCREEN initiative, implemented in 13 countries, has strengthened regional genomic surveillance through investments in sequencing infrastructure, workforce training, and cross-country coordination, evolving from a SARS-CoV-2-focused effort into a multi pathogen platform. By building local capacity, AFROSCREEN reduced dependence on external laboratories, helped close critical surveillance gaps, and generated genomic data that could inform mitigation of national health risks. However, persistent structural challenges related to inequity, including procurement delays, limited bioinformatics autonomy, reliance on short term funding, high staff turnover, and weak integration of genomic data into national decision making, continue to constrain its full potential, underscoring the need to embed genomics within national systems to advance genomic sovereignty and preparedness for future epidemics.

Genomic surveillance has become a cornerstone of public health, as demonstrated during the COVID-19 pandemic, where globally generated data enabled the swift development of diagnostics [1], guided vaccine design [2], and tracking how viral evolution impacts diagnosis [3], infectivity [4], virulence [5], and transmission dynamics [6], thus supporting timely containment efforts.

However, the pandemic also revealed stark inequalities in genomic surveillance capabilities worldwide, with most African nations lacking the tools and expertise required to detect emerging variants [7]. Early detection of highly transmissible and evolving variants like delta and omicron required robust genomic systems that were absent in many countries across the continent, as they were dependent on sequencing in the Global North or some hubs in the Global South. These disparities have fuelled growing debates on genomic sovereignty: the ability of countries to generate, analyse, and govern their own genomic data.

Recognising this equity gap, the AFROSCREEN project was established to strengthen local genomic surveillance capacity building on pre-existing partnerships between three coordinating institutions (*ANRS Maladies Infectieuses Emergentes*, *Institut Pasteur*, and the *Institut de Recherche pour le Développement*) and national laboratories and ministries of health in the region. Thirteen countries were included in the project on this basis: Benin, Burkina Faso, Cameroon, the Central African Republic (CAR), the Democratic Republic of Congo (DRC), Ghana, Guinea, Ivory Coast, Madagascar, Mali, Niger, Senegal, and Togo.

Aligning with the Pathogen Genomics Initiative led by the Africa Centres for Disease Control and Prevention (Africa CDC) and the International Pathogen Surveillance Network managed by the World Health Organization (WHO), the project aimed to tackle health inequities by prioritising genomic sovereignty, to meet immediate national public health needs and lay the groundwork for better epidemic preparedness across the continent.

## AFROSCREEN: A PROGRAMME FOR STRENGTHENING GENOMIC SURVEILLANCE INFRASTRUCTURE AND EXPERTISE IN RESEARCH

The AFROSCREEN project implementation followed a predefined logical framework that articulated objectives, activities, and expected outputs related to genomic surveillance capacity building. It began with a comprehensive assessment of laboratory capacities aimed at identifying gaps in equipment, resources, and technical expertise. To address these needs, the project implemented next-generation sequencing platforms adapted to local contexts, ranging from portable and cost-effective systems to higher-throughput instruments for better resourced laboratories, alongside essential laboratory equipment (nucleic acid extraction systems, PCR kits, cold storage, biosafety cabinets, sequencing reagents, and computing infrastructure for local analysis).

In parallel, a network of sentinel surveillance was expanded from 7 to 57 sites, embedding genomic surveillance within routine national monitoring of emerging infectious threats. Over 250 professionals were trained in epidemiological investigation, sequencing, and bioinformatics. Their training was adapted to the changing epidemiological landscape, enabling multi-pathogen sequencing capacity beyond SARS-CoV-2.

The AFROSCREEN programme fostered local expertise by linking 25 health and research centres, including regional reference laboratories, academic institutes, and hospital diagnostic centres. This grew out to a strong community of practice of shared learning and harmonisation of workflows in regular technical exchanges.

To strengthen the availability of genomic and epidemiological data, AFROSCREEN developed a secure project database and supported standardised monthly reporting on a secure collaborative platform. National laboratories were encouraged to deposit SARS-CoV-2 sequences in the Global Initiative on Sharing All Influenza Data (GISAID) repository, a surveillance platform recognised by WHO, to ensure international visibility, interoperability, and contribution to global genomic surveillance. At the same time, full sequencing results were first shared with the relevant national health authorities, which retained responsibility for determining whether and how data should be communicated more broadly.

## IMPACT ON SURVEILLANCE, RESEARCH, AND PUBLIC HEALTH

A continent-wide study showed that the number of African countries that can sequence locally was growing in 2022 and this was linked to a faster turnaround time (time between the date of sampling and to the date the resulting sequence is submitted to the database), making the data timely and instructive for public health actions [8]. Here we discuss the impact of the capacity building of AFROSCREEN specifically on public health in terms of contributions to closing surveillance gaps for SARS-CoV-2 and other emerging pathogens, the integration of genomic data with epidemiological and clinical information, and addressing knowledge gaps through research. Together, these advances increased the availability of genomic data for national public health interpretation, even when translation into actual policy remained variable.

AFROSCREEN helped implementing routine SARS-CoV-2 surveillance where it did not previously exist or was not operational. Studies from Burkina Faso, Cameroon, Niger, and Guinea reported genomic surveillance from periods or regions that had been largely absent from global datasets [9–13]. Additionally, post-epidemic sentinel surveillance in Guinea demonstrated that SARS-CoV-2 circulation persisted beyond the acute pandemic period [14]. Beyond documenting viral circulation, genomic data were linked with clinical data in Cameroon and with antibody titers in Guinea, supporting assessment of variant-specific and population-level public health risk [15,16]. National-level AFROSCREEN-generated data were integrated into continent-wide analyses, enabling comparison of local trends with broader patterns of SARS-CoV-2 spread across Africa [17]. Together, these findings illustrate that routine genomic surveillance can be implemented and sustained in resource-constrained settings to fill surveillance gaps to inform national public health risk assessment.

As the programme evolved, surveillance capacities initially established for COVID-19 were redeployed to other (re)emerging pathogens. This resulted in several genomic surveillance studies, including the first characterisations of circulating dengue viruses in Niger and Benin, confirming lineages in neighbouring regions were also present locally [18,19]. These experiences also informed the re-evaluation of syndromic sentinel surveillance systems, leveraging genomic capacity to strengthen epidemic preparedness, as illustrated by work on severe acute respiratory infections [20]. In Guinea, genomic investigations prompted a reassessment of syndromic surveillance assumptions by showing that a substantial proportion of measles-like rash cases were attributable to non-measles viruses, including rubella [21]. In the CAR, newly generated genomic data addressed a critical knowledge gap during a period of sustained human transmission of mpox in neighbouring countries, demonstrating predominantly zoonotic spread of mpox in the CAR and drawing attention to rural risk and unresolved questions around animal reservoirs [22]. Collectively, these studies show that newly operational platforms can be reused to prepare for and respond to evolving public health risks.

Extending this adaptability of the genomic platforms beyond human surveillance, one study examined the co-circulation of mpox and non-human pox viruses, illustrating the complexity of poxvirus reservoirs [23]. In a similar vein, another study identified hepatitis E in pigs as a known zoonotic reservoir in Guinea that had been under-characterised due to the lack of integrated animal-human surveillance [24]. Although these activities were not an initial objective of AFROSCREEN, they demonstrate the potential of genomics to address One Health questions directly relevant to public health priorities.

Another category of supported studies focused on evaluating diagnostic strategies under real-world constraints, sharing valuable lessons learned outside of surveillance or epidemiological research questions. This included the assessment of a widely deployed Chikungunya virus assay whose performance was compromised by misalignment with local viral diversity [25]. In settings where optimal samples are unavailable alternative approaches were explored, including the use of rapid diagnostic test strips as a source of RNA for sequencing [26] and the integration of serology to complement genomic analyses, enabling the identification of mpox outbreaks that would otherwise remain undetected [27].

Overall, these studies demonstrate that AFROSCREEN-supported genomic surveillance platforms can be sustained in resource-constrained settings and flexibly redeployed to address evolving public health questions, from routine monitoring to outbreak investigation and preparedness for emerging threats.

## FOCUS ON MPOX SEQUENCING IN THE DRC

The mpox outbreak in the DRC provides a concrete example of how AFROSCREEN-supported genomic capacity could be redirected to address an emerging public health priority. In the DRC, mpox sequencing was conducted in national laboratories whose genomic capabilities had been strengthened through AFROSCREEN and complementary capacity-building initiatives. Through provision of sequencing reagents, training on laboratory protocols, and support for bioinformatic analysis, AFROSCREEN enabled the integration of genomic data into ongoing epidemiological investigations aimed at characterising transmission patterns and viral diversity. The resulting studies substantially informed understanding of the outbreak and contributed to the evidence base considered by international public health bodies, including in the context of the declaration of mpox as a public health emergency of international concern in August 2024.

One key study documented, for the first time, the sexual transmission of clade I mpox [28], revealing an unexpected transmission route that challenged prevailing framing of clade I as zoonotic, with implications for risk communication and contact tracing strategies. Further research identified a novel mpox clade, clade Ib, in the eastern DRC [29], providing genomic evidence linking clade Ib to sustained human transmission in an endemic setting, signalling a heightened outbreak risk and need for adapted containment strategies. Nevertheless, focusing on only human transmission would be imprudent, as an analysis of 603 sequenced mpox cases revealed zoonotic transmission as the predominant driver of the virus’s genomic diversity in the DRC, highlighting the coexistence of zoonotic and human transmission pathways [30].

Building on these insights, genomic surveillance permitted the detection of co-circulation of clades Ia and Ib in Kinshasa [31], as well as the first imported clade Ib cases in Goma [32], enabling better case definition. Independent introductions of clade Ia from the CAR to the DRC were also reported, underscoring the need for regional surveillance [33].

Collectively, these studies demonstrate how in-country genomic surveillance can refine our understanding of pathogen transmission dynamics in near real time and support more targeted, context-appropriate public health measures.

## FIVE LESSONS LEARNED

While AFROSCREEN achieved significant milestones, it faced challenges that highlight the complexities of implementing genomic surveillance in resource-limited settings. These challenges and their associated recommendations were collaboratively identified during workshops and plenary sessions at a symposium involving all network members, as well as key national and international stakeholders, including representatives from national health ministries, WHO and Africa CDC. Lessons learned and recommendations described here were derived through continuous project monitoring using predefined indicators and periodic progress reports, findings from an independent external evaluation, and structured feedback collected during a regional end-of-project colloquium involving national health authorities, implementing partners, and scientific experts. These sources were used to identify recurrent challenges, enabling factors, and priority recommendations discussed in this article.

### Accelerate procurement and reduce customs delays

A recurrent challenge concerned infrastructure and logistics, particularly delays in procurement (*e.g.* purchasing negotiations with a major sequencing supplier stalled for several months) and customs clearance (*e.g.* reagents blocked at airport for several months), which significantly disrupted early project timelines. These delays reflected complex procurement and national import procedures that were not adapted to emergency genomic surveillance needs. These delays reflect structural inequities in the global genomics ecosystem and slow the ability to participate on equal footing in epidemic response. Procurement delays were a specific problem for purchasing partners that are constrained by public procurement rules, which can invoke lengthy procedures. When negotiations stalled during the omicron wave, emergency workarounds with freight forwarders mitigated some blockages, but did not completely prevent prolonged delays and generated additional costs.

In the second year of the project, a joint framework contract was created for equipment and consumables, a tender was launched, and a main freight forwarder was selected that was then navigating procurement across multiple countries. This procurement mechanism worked as intended, resulted in standardised supply and better navigation of national import procedures, but could not override custom systems. These experiences show that procurement and customs bottlenecks cannot be solved solely through *ad hoc* operational fixes during emergencies. Future programmes need to anticipate these at the designing phase by lining up multi-year procurement frameworks, and by seeking pre-negotiated arrangements with customs authorities to fast-track importation during emergencies.

### Simplify and standardise bioinformatics through ready-to-use pipelines

As sequencing activities intensified, limitations in data management and bioinformatics capacity became increasingly apparent. Storing, analysing, and interpreting large volumes of data proved challenging in many countries where bioinformatics expertise and infrastructure remain limited. While structured training can respond to immediate needs, one-off trainings alone are not likely to create robust capacity. In response, the project developed and deployed a ready-to-use genomic analysis pipeline (GeVarLi) [34] that was deployed across partner laboratories in Benin, Cameroon, Ghana, Guinea, Togo, the DRC, and Senegal. This approach reduced dependence on scarce bioinformatic expertise and shorten time from sequencing to actionable information. Wider adoption of standardised, user-friendly bioinformatic pipelines that allow laboratories to convert raw sequences to interpretable results with minimal manual coding is, therefore, likely to strengthen the impact of genomic surveillance. By reducing dependence on external expertise, this approach also addresses inequity by enabling countries to analyse and interpret their own data rather than relying on external partners, strengthening local ownership and visibility.

### Embed recurrent sequencing costs into national budgets and diversify partnerships

Beyond technical aspects, sustainability emerged as a central concern across discussions. Genomic sequencing entails high recurrent costs, including reagents, equipment maintenance, data infrastructure, and skilled personnel, making continued operation difficult when reliant on time-limited external funding. The AFROSCREEN programme successfully jump-started sequencing capacity, but dependence on a single donor represented a structural vulnerability. This was repeatedly raised by the implementation partners during the end-of-project colloquium, who warned that sequencing activity risked declining once project financing ended. The shared vision was that long-term viability depends on domestic resource mobilisation**,** with recurrent genomic surveillance costs embedded within national health or research budgets. To achieve this, genomic surveillance needs to be further institutionalised. Simultaneously, discussions underscored the importance of diversified funding, involving national (*e.g.* ministry of health), regional (*e.g.* Africa CDC), and international partners (*e.g.* WHO), as well as partners from the private sector. Together, these lessons point to the need for financing models that combine domestic resource mobilisation with coordinated partnerships, ensuring that sequencing capacity can be maintained beyond individual project cycles. Embedding recurrent costs in national budgets is, therefore, not only a sustainability measure, but an equity imperative that can reduce chronic dependence on external donors.

### Create career incentives to retain skilled staff

Another challenge that mostly became evident at the end of project was retaining skilled personnel. Many trained staff were employed through time-limited project funding, creating a risk that skills are lost once funding ends. Furthermore, while many people were trained in genomics and bioinformatics, institutions did not systematically offer clear career tracks for these skills. Without such pathways, African countries risk a continuous loss of locally trained talent to better-resourced institutions elsewhere, perpetuating global inequities in expertise and leadership in genomics. To address high staff turnover rates and ensure the continuity of laboratory operations, anchoring genomic surveillance posts within public health institutions is crucial, as well as career incentives and pathways to get recognised expertise in genomic surveillance and outbreak response. This would provide leadership roles that recognise expertise in genomic surveillance and outbreak response.

### Institutionalise genomic data flows into public health decision-making

Finally, effective collaboration of laboratories with public health authorities is essential for translating genomic insights into timely public health action and for ensuring financial sustainability (through dedicated national budget lines) and operational sustainability (through dedicated career pathways). Although AFROSCREEN strengthened the availability and interpretation of genomic data, its integration into national systems was initially limited, reflecting the emergency context in which the project was launched. Over time, repeated interactions between laboratories and surveillance actors took place. In some settings, sequencing results informed epidemiological interpretation (*e.g.* repeated family investigations in Burkina Faso were linked to laboratory results), situational awareness (*e.g.* the identification of circulating clades in the DRC), or technical discussions within surveillance units (*e.g.* in Cameroon, sequencing results were regularly discussed with clinicians). In other contexts, limited institutional linkages and the absence of formalised reporting mechanisms constrained uptake. Consequently, translation of genomic data into public health decision-making remained heterogeneous. This gap between data generation and decision-making underscores the need to move beyond technical capacity building and explicitly address institutionalisation. This entails clearly defined governance arrangements between sequencing laboratories, surveillance units, and ministries of health; standardised reporting pathways that embed genomic outputs within routine surveillance and alert systems; and structured interaction between laboratories and public health authorities to support joint interpretation and operational use of genomic data. Strengthening these institutional links is central to equity: it ensures that African-generated genomic data primarily serve national public health decision-making, rather than mainly feeding global surveillance systems.

## PERSPECTIVES

The next phase of genomic surveillance efforts in West and Central Africa will build on the recommendations from the end-of-project colloquium, ensuring alignment with local priorities and fostering Africa-led solutions. Efforts will focus on integrating genomic surveillance into national health strategies, strengthening regional collaboration, and building resilience against future health threats. The collaborations initiated by AFROSCREEN highlight the potential for reducing reliance on external actors; these partnerships should be deepened moving forward. Shared regional training programmes, coordinated outbreak responses, and joint research initiatives can foster ownership of surveillance activities across the continent.

Building on the first phase of AFROSCREEN, activities such as environmental monitoring and studies on zoonotic diseases are intended to be included, reinforcing the One Health approach and broadening the scope of genomic surveillance. The activities of the AFROSCREEN programme will remain flexible, adapting to new pathogens and emerging health threats, to ensure that genomic surveillance is always responsive to the evolving landscape of public health challenges.

Maintaining alignment with the Africa CDC’s Pathogen Genomics Initiative will be key to advancing these goals. Equally important is ensuring the incorporation of genomics into integrated disease surveillance at the national level, where sequencing data can inform decision-making alongside epidemiological, clinical and environmental indicators.

## CONCLUSIONS

Lessons learned from the AFROSCREEN project demonstrates that capacity building of genomic surveillance is not only a technical endeavour, but also a matter of health equity. By enabling in-country sequencing across 13 countries in West and Central Africa, the project reduced dependence on external laboratories, filled critical surveillance gaps for SARS-CoV-2 and other emerging pathogens, and improved the actionability of genomic data. At the same time, it revealed that technical capacity alone cannot resolve structural inequity challenges, including procurement bottlenecks, fragile data ownership, chronic underfunding, high staff turnover and difficulties translating genomic data into public health action. Based on these lessons, the AFROSCREEN consortium identifies five interdependent levers that must advance together: streamlined procurement frameworks, ready-to-use bioinformatic pipelines, sustainable financing embedded in national budgets, institutionalised links between laboratories and ministries of health, and stable career pathways to retain skilled professionals. Looking ahead, success will ultimately be measured by whether genomic data are generated locally, governed locally, and routinely used to inform national decision making.

## Additional material


Online Supplementary Document
