# Supplementary material for: Strengthening microbial genomics capacity in Africa for epidemic preparedness: key lessons from the AFROSCREEN programme
Source: J Glob Health. 2026 Apr 30;16:03012. doi: 10.7189/jogh.16.03012 (PMC13132006; doi:10.7189/jogh.16.03012)
Supplement: Online Supplementary Document [file jogh-16-03012-s001.pdf]

Supplement to: AFROSCREEN consortium. Strengthening microbial genomics capacity in Africa for epidemic preparedness: key lessons from the AFROSCREEN programme. J Glob Health. 2026;16:03012.

List S1. Full list of AFROSCREEN consortium members and their affiliations

Salissou Abdou Ahmed Mohamed, Centre Muraz (Burkina Faso); Steve Ahuka-Mundeke, Institut National de Recherche Biomédicale (Democratic Republic of the Congo); Prince Akil, Institut National de Recherche Biomédicale (Democratic Republic of the Congo); Yoan Alaho, ANRS Maladies Infectieuses Émergentes (France); Mathias Altmann, Université de Bordeaux (France); Raphael Amani, Institut National d’Hygiène Publique (Côte d’Ivoire); Marie Amougou Atsama, Centre de Recherche sur les Maladies Émergentes et Réémergentes (Cameroon); Adrienne Amuri Aziza, Institut National de Recherche Biomédicale (Democratic Republic of the Congo); Soa Fy Andriamandimby, Institut Pasteur de Madagascar (Madagascar); Reine Salomé Anguinze Yegbia, Institut Pasteur de Guinée (Guinea); Eloïc Lénox Atindegla, Institut de Recherche Clinique du Bénin (Benin); Ahidjo Ayouba, Institut de Recherche pour le Développement (France); Alexandre Azar, ANRS Maladies Infectieuses Émergentes (France); Aliou Barry, ANRS Maladies Infectieuses Émergentes (France); Dieynaba Barry, Centre Hospitalier National Dalal-Jamm (Senegal); Naima Barry, ANRS Maladies Infectieuses Émergentes (France); Alix Blevin, ANRS Maladies Infectieuses Émergentes (France); Isabelle Bonal, Institut de Recherche pour le Développement (France); Tara Brosschot, ANRS Maladies Infectieuses Émergentes (France); Valérie Caro, Institut Pasteur (France); Margaux Chavardes, ANRS Maladies Infectieuses Émergentes (France); Bakary Cisse, Centre Muraz (Burkina Faso); Fatoumata Cisse, Institut Pasteur de Guinée (Guinea); Adjo Seyram Comlan, Laboratoire de Biologie Moléculaire et d’Immunologie, Faculté des Sciences de la Santé, Université de Lomé (Togo); Cathy Sandra Goimelle Coti-Reckoundji, Institut Pasteur de Bangui (Central African Republic); Gilles Cottrell, Institut de Recherche pour le Développement (France); Caroline Coulon, Institut de Recherche pour le Développement (France); Anoumou Claver Dagnra, Laboratoire de Biologie Moléculaire et d’Immunologie, Faculté des Sciences de la Santé, Université de Lomé (Togo); Muriel De Souza, Centre Muraz (Burkina Faso); Eric Delaporte, Institut de Recherche pour le Développement (France); Koussay Dellagi, Institut Pasteur (France); Lamine Dia, Centre Hospitalier National Universitaire de Fann (Senegal); Ndongo Dia, Institut Pasteur de Dakar (Senegal); Moussa Moise Diagne, Institut Pasteur de Dakar (Senegal); Fatoumata Diallo, Centre Hospitalier National Universitaire de Fann (Senegal); Haby Diallo, Laboratoire de Biologie Moléculaire et d’Immunologie, Faculté des Sciences de la Santé, Université de Lomé (Togo); Mamadou Korka Diallo, Institut Pasteur de Dakar (Senegal); Maryam Diarra, Institut Pasteur de Dakar (Senegal); Fatoumata Diawara, Institut National de Santé Publique (Mali); Fatoumata Dicko, Centre Hospitalier Universitaire du Point G (Mali); Idrissa Dieng, Institut Pasteur de Dakar (Senegal); Seynabou Mbaye Ba Souna Diop, Institut National de Santé Publique (Senegal); Delia Doreen Djuicy, Centre Pasteur du Cameroun (Cameroon); Eric D'Ortenzio, ANRS Maladies Infectieuses Émergentes (France); Natasha Dubois Cauwelaert, ANRS Maladies Infectieuses Émergentes

(France); Ginette Elvine Edoul Mbesse, Centre de Recherche sur les Maladies Émergentes et Réémergentes (Cameroon); Marion Fanjat, ANRS Maladies Infectieuses Émergentes (France); Ella Cyrielle Farra née Gonofio, Institut Pasteur de Bangui (Central African Republic); Ousmane Faye, Institut Pasteur de Dakar (Senegal); Nicolas Fernandez-Nuñez, Institut de Recherche pour le Développement (France); Nadine Fievet, Institut de Recherche pour le Développement (France); Sandra Miriella Charlène Garba-Ouangole, Institut Pasteur de Bangui (Central African Republic); Thibaut Armel Chérif Gnimadi, Centre de Recherche et de Formation en Infectiologie de Guinée (Guinea); Célestin Godwe, Centre de Recherche sur les Maladies Émergentes et Réémergentes (Cameroon); Solène Grayo, Institut Pasteur de Guinée (Guinea); Emilande Guichet, Institut de Recherche pour le Développement (France); Sylvie Guillemaut, Institut Pasteur (France); Ibrehima Guindo, Institut National de Santé Publique (Mali); Juliette Hardy, Institut Pasteur (France); Alice Henry-Tessier, Institut Pasteur (France); Magali Herrant, Institut Pasteur (France); Parfait Houngbegnon, Institut de Recherche Clinique du Bénin (Benin); Roughyatou Ka, Centre Hospitalier National Dalal-Jamm (Senegal); Adèle Kacou-N'Douba, Université Félix Houphouët-Boigny (Côte d'Ivoire); Kadio Jean-Jacques Olivier Kadio, Centre de Recherche et de Formation en Infectiologie de Guinée (Guinea); Hervé Kadjo, Institut Pasteur de Côte d'Ivoire (Côte d'Ivoire); Aliou Kamissoko, ARCAD Santé PLUS (Mali); Mouhamed Kané, Institut Pasteur de Dakar (Senegal); Dramane Kania, Centre Muraz (Burkina Faso); Ouattara Yakoura Karidja, Institut Pasteur de Côte d'Ivoire (Côte d'Ivoire); Alpha Kabinet Keita, Centre de Recherche et de Formation en Infectiologie de Guinée (Guinea); Eddy Kinganda Lusamaki, Institut National de Recherche Biomédicale (Democratic Republic of the Congo); Stéphane Kouadio Koffi, Centre Hospitalier Universitaire de Treichville (Côte d'Ivoire); Kobina Amandze Adams Kofi, Centre Hospitalier Universitaire de Treichville (Côte d'Ivoire); Yekayo Bénédicte Kone-Dakouri, Centre Hospitalier Universitaire de Treichville (Côte d'Ivoire); Abla Ahouefa Esther Konou (AAEK), Laboratoire de Biologie Moléculaire et d'Immunologie, Faculté des Sciences de la Santé, Université de Lomé (Togo); Yao Rodion Konu, Laboratoire de Biologie Moléculaire et d'Immunologie, Faculté des Sciences de la Santé, Université de Lomé (Togo); Charles Kouanfack, Centre de Recherche sur les Maladies Émergentes et Réémergentes (Cameroon); Vincent Lacoste, Institut Pasteur de Madagascar (Madagascar); Adamou Lagare, Centre de Recherche Médicale et Sanitaire (Niger); Magali Lago, Institut Pasteur (France); Ramatoulaye Lazoumar, Centre de Recherche Médicale et Sanitaire (Niger); Yann Le Pennec, Institut Pasteur de Guinée (Guinea); Cheikh Loucoubar, Institut Pasteur de Dakar (Senegal); Estelle Madaha, Centre Pasteur du Cameroun (Cameroon); Martin Maidadi-Foudi, Centre de Recherche sur les Maladies Émergentes et Réémergentes (Cameroon); Aminata Maiga, Centre Hospitalier Universitaire du Point G (Mali); Christian Malaka, Institut Pasteur de Bangui (Central African Republic); Santou Mamadou, Centre de Recherche Médicale et Sanitaire (Niger); Alexandre Manirakiza, Institut Pasteur de Bangui (Central African Republic); Achille Massougbodji, Institut de Recherche Clinique du Bénin (Benin); Placide Mbala Kingebeni, Institut National de Recherche Biomédicale (Democratic Republic of the Congo); Maimouna Mbanne, Institut Pasteur de Dakar (Senegal); Aminata Mbaye, Centre de Recherche et de Formation en Infectiologie de Guinée (Guinea); Geraldine

Meyer, ANRS Maladies Infectieuses Émergentes (France); Sarah Michel-Anfray, Institut Pasteur (France); Serge Freddy Moukaha-Doukanda, Institut Pasteur de Dakar (Senegal); Clara Muller, ANRS Maladies Infectieuses Émergentes (France); Joyce Mwongeli Ngoi, West African Centre for Cell Biology of Infectious Pathogens (Ghana); Mouchedou Abdoukarim Naba, Direction des Laboratoires de Biologie Médicale (Burkina Faso); Ousseynou Ndiaye, Centre de Recherche Clinique de Fann (Senegal); Christelle Nikiema, Direction des Laboratoires de Biologie Médicale (Burkina Faso); Richard Njouom, Centre Pasteur du Cameroun (Cameroon); Odilon Paterne Nouatin, Institut de Recherche Clinique du Bénin (Benin); Justus Nsio, Institut National de Recherche Biomédicale (Democratic Republic of the Congo); Dieudonnée Ouedraogo, Centre Hospitalier Universitaire Yalgado Ouédraogo (Burkina Faso); Armelle Pasquet, ANRS Maladies Infectieuses Émergentes (France); Martine Peeters, Institut de Recherche pour le Développement (France); Julien Poublan, Université de Bordeaux (France); Nicole Prada, ANRS Maladies Infectieuses Émergentes (France); Claudio Raharinandrasana, Institut Pasteur de Madagascar (Madagascar); Vololoniaina Raharinosy, Institut Pasteur de Madagascar (Madagascar); Christian Ranaivoson, Institut Pasteur de Madagascar (Madagascar); Rindra Randremanana, Institut Pasteur de Madagascar (Madagascar); Tsiry Hasina Randriambolamanantsoa, Institut Pasteur de Madagascar (Madagascar); Rila Ratovoson, Institut Pasteur de Madagascar (Madagascar); Norosoa Razanajatovo, Institut Pasteur de Madagascar (Madagascar); Vincent Richard, Institut Pasteur (France); Pierre Roques, Institut Pasteur de Guinée (Guinea); Celine Rouger, ANRS Maladies Infectieuses Émergentes (France); Mounerou Salou, Laboratoire de Biologie Moléculaire et d'Immunologie, Faculté des Sciences de la Santé, Université de Lomé (Togo); Safietou Sankhe, Institut Pasteur de Dakar (Senegal); Rabiatou Sanogo, Institut National de Santé Publique (Mali); Yacouba Sawadogo, Centre Hospitalier Universitaire Sourô Sanou (Burkina Faso); Maud Seguy, ANRS Maladies Infectieuses Émergentes (France); Islamiath Setondji Kissira, Laboratoire de Fièvres Hémorragiques Virales (Benin); Emeline Simon, ANRS Maladies Infectieuses Émergentes (France); Rachida Tahar, West African Centre for Cell Biology of Infectious Pathogens (Ghana); Aristophane Tanon, Centre Hospitalier Universitaire de Treichville (Côte d'Ivoire); Jules Brice Tchatchueng Mbouga, Centre Pasteur du Cameroun (Cameroon); Mathurin Tejiokem, Centre Pasteur du Cameroun (Cameroon); Mame Salane Thiam, Centre Hospitalier National Universitaire de Fann (Senegal); Isaac Tiembre, Institut National d'Hygiène Publique (Côte d'Ivoire); Bachirou Tinto, Centre Muraz (Burkina Faso); Noël Tordo, Institut Pasteur de Guinée (Guinea); Abdoulaye Toure, Centre de Recherche et de Formation en Infectiologie de Guinée (Guinea); Coumba Toure Kane, Centre Hospitalier National Dalal-Jamm (Senegal); Isidore Traore, Centre Muraz (Burkina Faso); Noumou Yacouba Keita, Institut National de Santé Publique (Mali); Anges Yadouleton, Laboratoire de Fièvres Hémorragiques Virales (Benin); Brice Yambyo, Institut Pasteur de Bangui (Central African Republic); Abo Yao, Programme PAC-CI (Côte d'Ivoire); Guillaume Zamina, Institut National d'Hygiène Publique (Côte d'Ivoire); Adama Zida, Centre Hospitalier Universitaire Yalgado Ouédraogo (Burkina Faso); Arsene Zongo, Centre Muraz (Burkina Faso).
